# Autogenerator-Based Modelling Framework for Development of Strategic Games Simulations: Rational Pigs Game Extended

**DOI:** 10.1155/2014/158679

**Published:** 2014-08-31

**Authors:** Robert Fabac, Danijel Radošević, Ivan Magdalenić

**Affiliations:** Faculty of Organization and Informatics, Pavlinska 2, 42000 Varaždin, Croatia

## Abstract

When considering strategic games from the conceptual perspective that focuses on the questions of participants' decision-making rationality, the very issues of modelling and simulation are rarely discussed. The well-known Rational Pigs matrix game has been relatively intensively analyzed in terms of reassessment of the logic of two players involved in asymmetric situations as gluttons that differ significantly by their attributes. This paper presents a successful attempt of using autogenerator for creating the framework of the game, including the predefined scenarios and corresponding payoffs. Autogenerator offers flexibility concerning the specification of game parameters, which consist of variations in the number of simultaneous players and their features and game objects and their attributes as well as some general game characteristics. In the proposed approach the model of autogenerator was upgraded so as to enable program specification updates. For the purpose of treatment of more complex strategic scenarios, we created the Rational Pigs Game Extended (RPGE), in which the introduction of a third glutton entails significant structural changes. In addition, due to the existence of particular attributes of the new player, “the tramp,” one equilibrium point from the original game is destabilized which has an influence on the decision-making of rational players.

## 1. Introduction

This paper discusses the issue of simulation of strategic games that fall within the domain of game theory analysis. As a discipline of interactive decision-making, game theory deals with noncooperative as well as cooperative scenarios. However, game theory is primarily concerned with analyzing competitions or conflict situations, defining the choices for each individual player and examining possible resulting outcomes and behaviors in such competitive games [1]. Deeper insights into the established game theory scenarios or models have been enabled by the application of computer programs. Yet tractability limitations have been found to be a common disadvantage of computer-performed simulation models [2]. Namely, the decision process is hard to trace even if the source code is provided. The modelling framework for the development of strategic games scenarios described in this paper gives a detailed view of the tool on which the simulation is performed and thus increases the tractability of simulations.

A literature review reveals that common approaches in the field of game theory are the use of agents [3] and different models of learning such as the Monte Carlo method and temporal difference learning [4]. The intention of our approach entirely focused on real players is to provide a modelling framework that would be easily upgradable with new features. Our core example refers to performing a simulation of the well-known game from the theory of games called “Rational Pigs” or “Boxed Pigs.” The basic scenario examines strategic solutions for two gluttons (two pigs) that are placed inside a large cage where each of them can use a lever to cause the release of food into the cage [5]. One of the gluttons is relatively large, which brings asymmetry into the strategic situation. The food is released into the cage at the opposite side of the lever, which presents a handicap for the glutton who actually presses the lever because of the delay in his feeding activity. Although rational decision-making in the afore-described situation has been analyzed by a number of authors such as [6–8] and others, the most acknowledged analysis is generally considered to be the one by McMillan [9]. Gintis in [10] discusses a game which involves a large and a small monkey, where the strategic framework is almost identical since it is necessary for the provision of food to climb a tree with fruits and shake the tree.

The particular feature of the approach presented in this paper is that in it generative programming techniques are applied to the “Boxed Pigs” model. According to Czarnecki and Eisenecker [11], generative programming is a discipline within automatic programming aimed at automating the software development process. In general, such automatically produced software is stored for later use (e.g., in program files). On the other hand, in the autogenerator-based approach [12] the necessary piece of code is produced and immediately executed on demand. Instead of program files, in the case of autogenerator the generated code is stored into variables and also executed from them. The concept also uses the convenient capability of scripting languages to autoevaluate the programming code. In our particular case Python is used owing to the flexibility of its supported data structures (e.g., Python lists) and its object model that is much more sophisticated in comparison with other scripting languages oriented to string processing (e.g., Perl or JavaScript).

In the simulation of the elaborated strategic game, program specification is used for code generation as well as for recoding a set of game properties in course of the game. Such properties can refer to the game as a whole (like the state of a semaphore and the game phase) and tanks (their position, amount of fuel, open or closed state, etc.) as well as to players (position, chosen tank, amount of fetched fuel, game restrictions, etc.).

In addition to applying generative programming techniques, in the presented research we focused on the formulation of more complex scenarios through “Rational Pigs Extended,” where the game is modelled with three gluttons, in order to expand the sphere of research into this matter. The first issue examined concerns the stability of game solutions considering two gluttons in a new situation that includes a third player. The second important issue refers to the possibility of experimental laboratory examination of the players' learning that occurs in repeated game scenarios.

## 2. Modelling a Framework for the Development of Games of Strategy

### 2.1. Introduction to Autogenerator

Autogenerator is a model of application development where program code is generated and executed on demand. It is based on the SCT generator model [13] that is aimed to produce entire applications instead of skeletons that require additional work.

**Figure 1:**
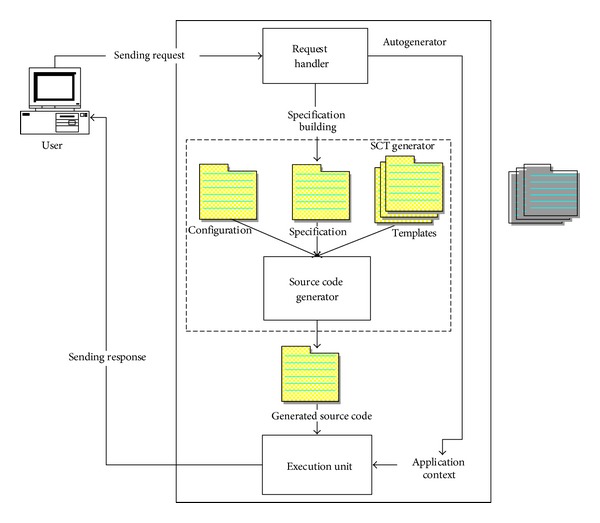
The autogeneration process.

Autogenerator represents an example of the advanced use of frame-based software development. Unlike some other frame-based generator models, like XVCL [14] or Bassett's frames [15], autogenerator relies on dynamic frames, which means that frames are dynamically created during the source code generation process. This increases flexibility in the development of generators with regard to the use of static frames. In the acronym SCT the basic model elements—Specification (the term* Specification* (written with “S”) refers to the model element of the SCT generator model), Configuration, and Templates—are comprised.* Specification* contains user/developer specified features of the generated application in form of attribute-value pairs.* Configuration* is a set of rules that manage the generation process, where programming code is produced by assembling the features from* Specification*, together with code artifacts stored in* Templates*.* Templates* refer to a set of code artifacts that are used as building blocks of generated applications.* Specification*,* Configuration,* and* Templates* together build an SCT frame, which contains all the information needed by the SCT generator to produce program code. The autogeneration process is shown in Figure 1. The user sends a request which contains information about the user and the action that the user wants to take. The request is accepted by the request handler, whose task is to decompose the request, determine what action to take, and call the source code generator to produce the appropriate source code. It should be noted that in the autogeneration process only the source code that is needed for the user's request to be fulfilled is generated. The generated source code is then stored in a variable, where it can be evaluated by scripting languages like JavaScript, Perl, or Python. The generated source code is evaluated by the execution unit, as shown in Figure 1. The execution unit executes the generated source code together with the arguments provided by the request handler. Those arguments are represented in Figure 1 as the application context. The execution unit sends the result to the user.

In the former version of autogenerator three key new features of generated software were introduced. The first one is the possibility of changing the application “on the fly,” which means that any change in* Specification* is immediately applied to the autogenerated application. The second one is the usage of imperative instructions in* Specification*. Such instructions are aimed to be performed only once, usually to harmonize the program code with some program dependency (e.g., to perform the ALTER TABLE instruction to change the database table structure when the user/developer adds/deletes some field in* Specification*). After they have been executed, imperative instructions are deleted from* Specification*. The third feature is introspection which, in the case of autogenerator, gives the developer an insight into basic model elements that are used in the production of a specific piece of code (e.g., whose subset of the SCT model is used in the production of some data editing form, data review, etc.).

The model of autogenerator was extended for the purpose of implementing a simulation mechanism, like the strategic game in our example, by introducing the possibility of changing* Specification* from the autogenerated application. This includes operations such as changing attribute values, adding new attributes, deleting the attributes, and testing their values. Each change in* Specification *changes the generated code, making the entire application dynamic. All the aforementioned features are applied in the program example.

### 2.2. Autogenerator in Strategic Games

**Figure 2:**
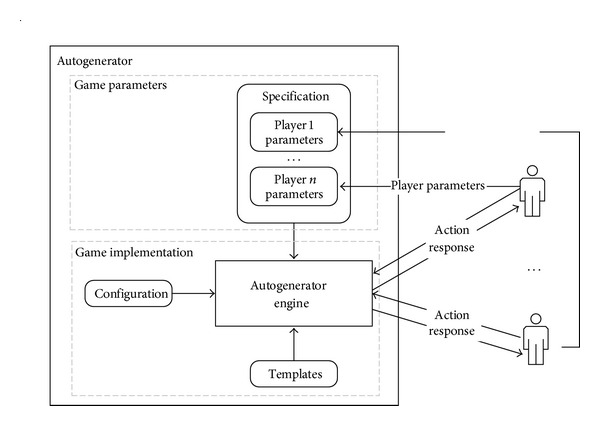
Model of using autogenerator in strategic games.

The model of the usage of autogenerator in strategic games is presented in Figure 2. The implementation of the game is performed by means of configuration and templates of the program code. Each strategic game has its own configuration and its own set of program code templates. The configuration defines the way in which program code templates are combined to produce the final program code. If a new feature is to be introduced into a strategic game, new templates will have to be created and the configuration will have to be updated. The introduction of new features is possible even if a strategic game is running since autogenerator produces program code on demand. The possibility of changing the rules in a game that is running is thus an important feature of using autogenerator in strategic games.

The number of players and their parameters can change over time since they represent real-life scenarios. The number of players and their properties are defined in* Specification* by a set of parameters. Each player has their own set of properties that can be updated, extended, or reduced during the game. Since some strategic games can run longer, adding new players and changing their properties during the game make for another key feature of autogenerator usage in the context of strategic games.

The implementation of the presented model of autogenerator in strategic games is described in detail on the model of the Rational Pigs game discussed in Section 4.

## 3. Extended Autogenerator Model 

Although the feature of regenerating program code on demand was already included in the original model of autogenerator presented in [12], the possibility of changing its* Specification* by the autogenerated application (in form of imperative instructions, as described below) was limited. To fulfill the requirements of different simulations, it would be useful to specify the attributes of simulated objects in* Specification* and changing values of its attributes during execution. The updated attributes and their values could be used in the following regeneration cycle in production and execution of the new program code (Figure 2). Such frequent reading and updating of* Specification* may give rise to some implementation issues, especially in the case of concurrent access by several autogenerated processes, as discussed in Section 3.2.

On the other hand, the possibility of updating* Specification* can eliminate the need for usage of any external data sources within simulations like strategic games.

### 3.1. Updating Specification

**Figure 3:**
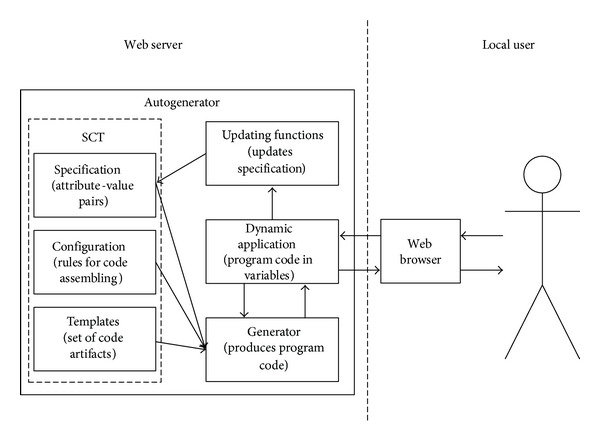
Updating attributes and their values.

As shown in Figure 3, the user's Web browser communicates with the dynamic application that is created on demand by the generator. The demand can be sent by the user (e.g., by clicking on a link or a button) or periodically, to refresh the state (e.g., the state of a simulation, according to states of participating objects).

Updating functions enables changes in* Specification* concerning attribute values and adding/deleting attributes. It is also possible to check the existence of an attribute and its value. There are five updating functions in the current model (Table 1).

By using updating functions,* Specification* also assumes the role of a small database that can be used in further generation of the code and the report that contains the values obtained during execution.

### 3.2. Design and Implementation Issues

Two main issues occurred during the process of designing updating functions. The first one concerns addressing the attributes in* Specification*. Although* Specification* has a tree structure, which suggests the usage of paths similarly to addressing folders on a hard disk, such paths can be long and should be changed in case of tree restructuring. In the approach presented in this paper, names and values of parent attributes are therefore used instead of paths, with the assumption that their names are unique. Taking into account this limitation, it is simple to address parts of* Specification* as in the following example:

 
*fuel = float(* ***read_value*** *(“+connected_tanks”, “yes”, “++fuel”, “none”))*
 
***update_value*** *(“+connected_tanks”, “yes”, “++fuel”, str(fuel-1))*

which reads and updates the value of* fuel *(decreased by 1) in* Specification* as follows:

 
*common:*
 +*semaphore:red*
 +*game_start:yes*
 +*reset:yes*
 
**+*connected_tanks:yes***   <- parent attribute/value
 
**+*+fuel:60*** <- target attribute/value
 …

Another issue that was encountered concerns concurrent access to* Specification* by more autogenerated processes, for example, in simulations with more (relatively) independent actors, players, or objects. In some cases,* Specification* was reached by a process at the moment when it was only partially updated, which led to a temporal collapse of the autogenerated application.

For the purpose of the example implementation, a simple system of locking/unlocking of* Specification* was established to avoid conflicts. Each updating function deals with* Specification* in the same way:

 
*Wait to be unlocked/timeout passed*
 
*Lock Specification*
 …
 
*Perform update*
 …
 
*Unlock Specification*

Although this solution could diminish the performance of the autogeneration system, no such effect was noticed in the example application.

### 3.3. Usage of Extended Autogenerator Model in a Strategic Game Example

**Figure 4:**
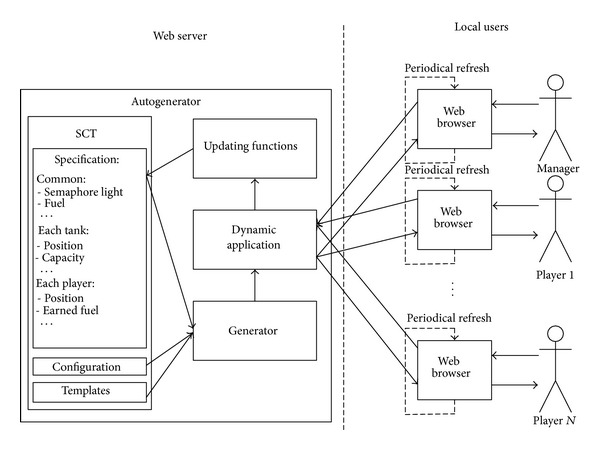
Extended autogenerator model in a strategic game example.

The strategic game example (available online at http://gpml.foi.hr/Autogenerator_Strategic_Game/) includes several players, depending on* Specification*. Along with them, there is a special player—the “manager,” who manages the strategic game by choosing the game scenario, starting a new game, giving permission for opening tanks (in form of a green light on the semaphore), and giving permission for motion to tank and earning fuel (also in form of a green light on the semaphore). As shown in Figure 4, each player accesses the dynamic application which is produced by autogenerator on demand. This demand could be given directly by the player (e.g., by a button click), but there is also a periodical refresh (every 1.5 seconds in the example) that invokes autogenerator and serves for showing the updated state of the game (positions of players, states of tanks and fuel, etc.).

During the game, the values of the attributes in* Specification* change, reflecting the actual state. In addition, values achieved during the simulation can be easily read from* Specification*.

## 4. Modelling Rational Pigs Game

The situation referred to as a “Rational Pigs scenario” is well known in game theory. Consideration of rationality in this game has its roots in the famous experiment from 1979, reported by Baldwin and Messe, wherein the intelligence of animals was tested [16]. On the example of the strategic game in Table 2, we analyze the decisions of participants and search for an equilibrium state, that is, the solution to the game.

While some authors suggest payoffs that are somewhat different from those shown above, making generalizations as in [17], others reduce the importance of the effort and the associated cost due to the pressing of the lever, as discussed in [6], which practically leads to possible negative payoff for** Sp**. Therefore, in our example the amounts of the payoffs matrix are somewhat loosely defined as

(1) $$\begin{array}{c} \left(\begin{array}{cc} 1.5;4.5 & 1,5\\ 3,3 & 0,0 \end{array}\right). \end{array}$$

The solution to the presented game is calculated using the procedure of Nash equilibrium, using arguments proposed in [18]. For* n*-player game in a strategic (matrix, normal) form* G* the defined set of players *I* = {1,…, *n*}, where “−*i*” stands for a set of *I*∖{*i*}. The utility that a* player i* realizes in the game is denoted by *u*_*i*_. The space of pure strategies of* player i* is denoted by *S*_*i*_. The Nash equilibrium s* is defined, according to [19–21], so that

(2) $$\begin{array}{c} u_{i}\left(s_{i}^{*},s_{-i}^{*}\right)\geq u_{i}\left(s_{i},s_{-i}^{*}\right),\;\forall i\in I,\;\;\forall s_{i}\in S_{i}. \end{array}$$

The Nash equilibrium thus represents a set of players' best responses to their opponents' strategies. Important theoretical and behavioural questions about the rationality of players emerge in practical analogies of such scenarios. Analysts such as McMillan [9] examined the logic of **Dp** through the analogy with strategic scenarios of a real-life cartel (OPEC), where Saudi Arabia assumed the role of the dominant glutton. In the repetitive scenario of this game, the existence of mixed Nash equilibria is possible, with the coresponding rationality of choosing probabilities (frequencies) of strategies. We anticipate this game as a one-stage game.

Since model (1) relativizes the cost of effort regarding pressing the levers in the game scenario, along with the existing solution to the game (DP, P) (Table 2), in accordance with (2), there is another Nash equilibrium for the two strategic choices (P, DP). In order to perform the simulation, we transform the payoffs matrix in the form of (multiplied)

(3) $$\begin{array}{c} \left(\begin{array}{cc} 15,45 & 10,50\\ 30,30 & 0,0 \end{array}\right). \end{array}$$

Furtherhermore, in the game shown in (3) we find two pure Nash equilibria: NE1 for strategies (DP, P) with payoffs* u*(NE1) = (30,30) and NE2 for strategies (P, DP) with payoffs* u*(NE2) = (10,50).

**Figure 5:**
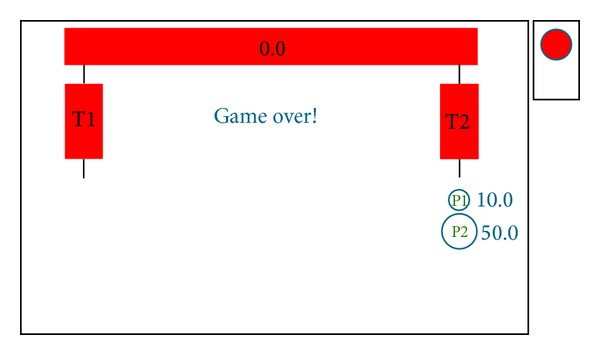
Rational Pigs outcome (*Press-Don't press *choices).

The outcome of the simulation for players' choices (P, DP), performed with the aid of autogenerator and with specific attributes of players** Sp** and** Dp **(*v*_1_, *v*_2_, *s*_1_, *s*_2_) given in Table 4, is shown in Figure 5. The set of payoffs in matrix (3) above is achieved in simulations, with the attributes of the two gluttons defined as in Table 4.

## 5. Rational Pigs Game Extended (RPGE)

Although the Rational Pigs framework provides a specific insight into decision-making dilemmas and allows for consideration of the rationality of strategic interaction between two participants, for the purpose of processing scenarios with a higher complexity we created a version of the game entitled “The Rational Pigs GameExtended”* (RPGE)*. Analyses of rationality overtly linked to the RPG scenario have been recorded with regard to issues related to investment and innovation supporting as well as artificial intelligence concepts, in [22–24]. The RPGE framework is supposed to contribute to the acquisition of knowledge regarding the topics listed.

The game is enriched by an additional glutton—“tramp,” which is always positioned in the middle of the cage at the beginning of the game. The original frame is thus changed to include the following new features:

- in the larger cage there are two feeding mechanisms situated opposite each other as well as two levers;
- in the game there are already more than two gluttons, so the extended primary scenario considers the interaction among three (3) gluttons;
- the gluttons are described in terms of the speed of movement, the speed of feeding (proportional to size), and their position;
- the two gluttons are basically static, located near their respective levers and feeders, and can press the lever;
- the small glutton moves faster than the large one;
- one (new) glutton can be denoted as a “tramp,” being positioned approximately in the middle of the cage, at an equal distance from both sources of food; he moves at a high speed while his size (speed of feeding) varies for the purpose of the experiment;
- the new glutton has no ability to press a lever that releases food into the cage, but when feeding areas are opened, he goes to pick up his portion;
- in the case that both feeding areas T1 and T2 are opened, the new glutton will hesitate for a while before heading to one end of the cage for food (choosing either left (T1) or right (T2)).

In the simulation performance, the available strategies in the formulated context of the game actually contain two steps: choice (P or DP) and moving towards the source of food. The motion itself is unified with the first decision. Regarding the third player, we discuss his movement (to the left or to the right) as his entire strategy. Although in the case of participation of three players the definition of Nash equilibrium becomes more demanding, the question of participants' rational behavior is even more prominent. Namely, players who achieved two Nash equilibria in a set that does not include a third glutton now need to review their previous optimally stable outcomes. A part of the earlier achievements of the two players** Sp** and** Dp** (portions of food labeled *ε* and *δ*) are transferred to the “tramp”** Tp** (Table 3). The values of *ε* and *δ* depend on the** Tp** attributes, that is, the speed of movement, speed of feeding (size), delay in reaction (in case of [open, open] situation), and the opening moves played by** Sp** and** Dp**.

We maintain that the conditions for a Nash equilibrium game in a two-player scenario will remain the same in the three-player game. If we assume that *ε*, ε*, *δ*, δ* > 0, as a condition for the stability of equilibrium NE1 = (DP, P, L), we obtain

(4) $$\begin{array}{c} 30-\varepsilon_{1}>15-\varepsilon_{1}^{*};\;\;30-\delta_{1}>0. \end{array}$$

Based on the above, the three-player equilibrium is NE1 = (DP, P, L), with payoffs (30 − *ε*_1_; 30 − *δ*_1_, *ε*_1_ + *δ*_1_). Furthermore, the required conditions associated with Nash equilibrium NR2 = (P, DP, R)

(5) $$\begin{array}{c} 50-\delta_{2}>45-\delta_{2}^{*};\;\;10-\varepsilon_{2}>0 \end{array}$$

which defines another Nash equilibrium for the three-player scenario, with strategies NE2 = (P, DP, R) and payoffs (10 − *ε*_2_, 50 − *δ*_2_, *ε*_2_ + *δ*_2_).

## 6. Implementation of Rational Pigs Extended Using Autogenerator

After creating a three-player model, we performed several experiments varying the attributes of the** Tp** player. In conditions where** Tp** is comparable to** Sp** in terms of, for instance, his size, regardless of his speed, the logic of the game for the two main players does not change, and the solutions will remain in the two sets of strategies, as in the previous scenario. However, we found the** Tp** attributes which ensured the values of parameters *ε*, *δ* that disturbed the NE2 equilibrium.

**Figure 6:**
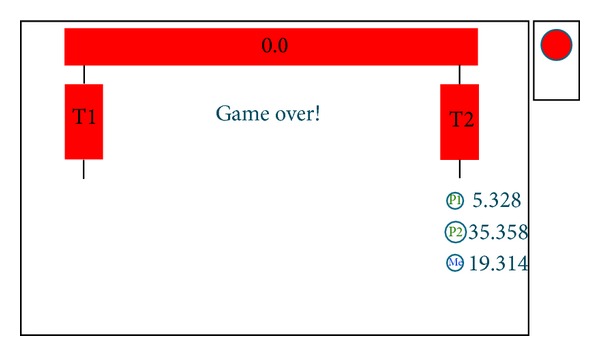
Rational Pigs Extended outcomes for (*Press, Don't press, R*) choices.

The outcome and realized payoffs for simulation with the delay parameter of 10 time units due to hesitation of** Tp**, for choices of strategies (P, DP, and R), and with other relevant data as in Table 4, are shown in Figure 6. In this case, analyzing conditions (5), it can be concluded that, for a sufficiently large (and fast)** Tp** player, the values of expressions (50 − *δ*_2_) and (45 − *δ*_2_*) became comparable, which caused a destabilization of Nash equilibrium NE2.

Payoff to the** Dp** player (Table 5) for particular chosen strategies (P, DP, and R) becomes comparable to or smaller than the payoff to outputs (P, P, and R) and (P, P, and L). Furthermore, for** Dp **strategy P is closed now to dominate over his strategy DP. In such a situation, the** Dp** player has doubts regarding employing his DP strategy since the optimal playing recommendations for the gluttons have changed.

Researching strategic decisions and achievements of players in borderline situations (Table 5) satisfying conditions (5) presents a challenge because it focuses on real players' rationality and possible behaviour in simulated conditions.

## 7. Discussion

We showed that autogenerator can be successfully used for the purposes of modelling and simulating (game theory) strategic games. In our example, autogenerator is implemented in a “Rational Pigs” strategic game and its created derivative entitled “RPG Extended.” In the research presented in this paper we realized the RPG model and enriched it by introducing a new player whose attributes may lead toward destabilization of the Nash equilibrium. This means that rationality previously found in the framework of a two-person game is no longer applicable.

Furthermore, this innovation enables us to consider strategic situations of a higher level of complexity, which are more appropriate for describing certain real-life scenarios. Therefore, future research may be focused on laboratory testing of the behavior of players in the roles of gluttons regarding their understanding of the change of circumstances resulting from the introduction of a “tramp,” their speed of response in terms of changing their preferred strategies, and so forth. This approach also enables the analysis of complex scenarios of RPGE at a given stage as well as the dynamics of the game by allowing for the same or similar scenario to be simulated or repeated over time. The game mechanism is prepared for laboratory testing by iterating the selected game scenario an arbitrary number of times, which includes both game variants (for two or three players). In this case game repetition is executed with the support of a central coordinator, thus enabling for the extensive form game to be performed that belongs to the class of finitely repeated games.

These games are generated by repetitions of a one-stage basic game, as described in detail in several sources, such as [10, 21, 25] and others. Participation in a repeated (iterated) game may result in higher payoffs than those achieved in a basic one-stage game [26]. The strategic profile of a particular player in a repeated game is placed in the so-called subgame perfect equilibrium, provided that the player has chosen a strategy of equilibrium in every contained subgame [27, 28]. According to the first Folk theorem proposed by Friedman in [29], any payoff in a one-stage game that dominates the Nash equilibrium, according to Pareto dominance, can be supported in a subgame perfect equilibrium for rational and sufficiently patient players. A subgame perfect equilibrium of a discounted repeated game with perfect monitoring is possible for a certain strategic profile, even if the corresponding payoff (P) does not dominate any one-stage NE, as stated by Fudenberg and Maskin [30] and further interpreted in [10, 25]. Nevertheless, such a scenario requires a high coordination level and adherence to an elaborate system of punishment of deviant players, among others [10, 31]. The deficiency of game theory concepts is that they generally allow for the existence of a large number of subgame perfect Nash equilibria, failing to achieve convergence, that is, targeting a small(er) number of solutions in repeated games. When predicting strategic behavior is concerned, gaming in laboratory conditions highlights the issue of participants' consistency and their motivation with regard to the actual perception of benefits on the one hand and the achievement of not-so-important points in a game on the other. It is noteworthy that some reports of the outcomes of experimental games (e.g., [32]) reveal significantly better results achieved by certain players in playing repeated games compared to theory-based expectations.

The players' interactions in a multistage game are interesting from several other perspectives, one of which is the scenario in which an individual glutton has limited information about his opponents, that is, no accurate knowledge of their relative speed, size, and other parameters. As a result, the behavior of players can be examined through the prism of games with incomplete information, as suggested by Gibbons [25]. By analyzing the players' behavior over time in multistage (RPG) games, we could focus on their learning and possible progress toward achieving better average payoffs. In conditions of varying degrees of the players' awareness, as previously discussed in the works of Camerer et al. in [19, 33], indicators of learning (consideration, commitment, and change) contained in the so-called EWA (experience-weighted attraction) model can be identified. Alternative attempts of establishing learning styles and learning successfulness using experimental methods, conducted in correspondence with the EWA model, were reported by some authors, such as [34].

## 8. Conclusions

The paper discusses the usage of autogenerator in the domain of computer simulation. It has been verified in the example of the strategic game RPG (E). The presented approach offers new possibilities in comparison with classic approaches of simulation of (live) agents' interactions. There are several benefits provided by the usage of autogenerator in strategic game simulations. Autogenerator introduces flexibility in specifying game parameters before the game is started, as well as after the game has been running for some time. Furthermore, autogenerator enables the performance of an arbitrary number of game iterations belonging to the same game scenario with monitoring of players' behavior. All changes in the game are saved in* Specification*, including the state at the end of the game. At the same time, all changes in* Specification* have their impact on dynamic code generation and, consequently, the execution of the game. For this purpose, the original autogenerator model was extended to enable* Specification *updates. This flexibility includes the specification of game parameters, which consist of variations in the number of simultaneous players and their features and game objects and their attributes as well as some general game features.

The aforementioned features of the autogenerator-based simulation system were used in the implementation of the “Rational Pigs” game (RPG). We showed that the values of players' payoffs previously recorded in the literature, such as those by McMillan [9] or Rasmusen [6], can be obtained if their players' attributes are found at appropriate values. In addition, by introducing a third glutton, “the tramp,” we created a new scenario entitled “RPG Extended” that can change the points of Nash equilibrium. This finding highlights some new issues in conceptualizing the rational behavior of players and opens up a possibility of further theoretical and experimental research, especially into related repetitive strategic scenarios.

Further benefits of such implementation of autogenerator may lie in simulation scenarios similar to RPGE found in various interactive organizations as well as the corporate sector, as emerging “serious games,” according to [35]. Possible examples include using the strategic management concept of “competitive dynamics,” where the firm's action or strategy is understood as a specific visible competitive move which, according to Chen [36], is initiated with the intention of enhancing the relative competitive positions. In addition, considering the value of the gaming approach within contemporary learning theories [35], including the theory of Cognitive Apprenticeship [37], and given the definition of effective learning, gaming as an activity could provide support in solving real-world problems in a relevant context and also facilitate the acquisition of required specific skills. In this way, it is possible to further develop the approaches presented in this paper in developing learning solutions for the corporate sector.

## Figures and Tables

**Table 1:** Updating functions.

Function	Arguments	Description
*try_attrib *	*attrib1* = parent attribute *value1* = value of parent attribute *attrib2* = target attribute	Checks the existence of a specified attribute in *Specification *

*read_value *	*attrib1* = parent attribute *value1* = value of parent attribute *attrib2* = target attribute *value2* = default function value∗	Reads the value of a specified attribute

*update_value *	*attrib1* = parent attribute *value1* = value of parent attribute *attrib2* = target attribute *value2* = new attribute value	Updates the value of a specified attribute

*update_attrib *	*attrib1* = parent attribute *value1* = value of parent attribute *attrib2* = old attribute name *attrib3* = new attribute name	Updates the name of a specified attribute

*update_add_value *	*attrib1* = parent attribute *value1* = value of parent attribute *attrib2* = name of the new attribute *value2* = value of the new attribute	Adds the new attribute and value to *Specification *

*update_delete_value *	*attrib1* = parent attribute *value1* = value of parent attribute *attrib2* = name of attribute to be deleted *value2* = value of attribute to be deleted (condition to be fulfilled for deletion)∗∗	Deletes the attribute and value

*If the target value cannot be found.

∗∗Can be replaced by an empty string (= no condition).

**Table 2:** Boxed Pigs model, as in [9].

	Strategies	Dominant pig (Dp)	
		Press (P)	Don't press (DP)
Subordinate pig (Sp)	Press (P)	1.5; 3.5	−0.5; 6
	Don't press (DP)	5; 0.5	0; 0

**Table:** (a)

Tramp Tp: left (T1)	Strategies	Dominant pig Dp	
		Press	Don't press
Subordinate pig Sp	Press	15 − *ε*_1_*; 45 − *δ*_1_*; *ε*_1_* + *δ*_1_*	10; 50; 0
	Don't press	30 − *ε*_1_; 30 − *δ*_1_; *ε*_1_ + *δ*_1_	0; 0; 0

**Table:** (b)

Tramp Tp: right (T2)	Strategies	Dominant pig Dp	
		Press	Don't press
Subordinate pig Sp	Press	15 − *ε*_2_*; 45 − *δ*_2_*; *ε*_2_* + *δ*_2_*	10 − *ε*_2_; 50 − *δ*_2_; *ε*_2_ + *δ*_2_
	Don't press	30; 30; 0	0; 0; 0

**Table 4:** Players' attributes resulting with NE destabilization.

Attributes	P1 (Sp)	P2 (Dp)	P3 (Tp)
Speed (*v*)	3*v*_0_	*v*_0_	6*v*_0_
Size (*l*)	*l*_0_	3*l*_0_	2*l*_0_
Delay (*T*); at (open, open)	0	0	10*T*_0_

**Table 5:** Rational Pigs Extended—payoffs according to the simulation with defined parameters (Table 4).

Tramp Tp (L)/(R)	Strategies	Dominant pig Dp	
		Press	Don't press
Subordinate pig Sp	Press	11,7 35,0 13,3 (L) 12,0 35,4 12,7 (R)	5,3 35,4 19,3 (R)
	Don't press	20,6 1,4 38,0 (L)	0; 0; 0
